# Circular RNA circ_0000518 promotes breast cancer progression through the microRNA-1225-3p/SRY-box transcription factor 4 pathway

**DOI:** 10.1080/21655979.2021.2019877

**Published:** 2022-02-03

**Authors:** Kang Shujuan, Li Zhongxin, Ma Jingfang, Cui Zhili, Wei Wei, Qian Liu, Yan Li

**Affiliations:** a Department of Brest, Affiliated Hospital of Hebei Engineering University, Handan, Hebei, China; b Department of Hepatobiliary Surgery, Handan Central Hospital, Handan, Hebei, China; c Department of Pathology, Affiliated Hospital of Hebei Engineering University, Handan, Hebei, China; d Department of Gynecology, Affiliated Hospital of Hebei Engineering University, Handan, Hebei, China; e Department of Oncology, The Second People’s Hospital, Dongying, Shandong, China

**Keywords:** Circ_0000518, miR-1225-3p, SOX4, BC

## Abstract

This work is designed to probe the functions and mechanisms of circ_0000518 in breast cancer (BC). qRT-PCR was performed to evaluate the circ_0000518, miR-1225-3p and Sry‑Related HMG box 4 (SOX4) mRNA expression in BC tissues and cells. After circ_0000518 was overexpressed in MDA-MB-468 cells, and circ_0000518 was knocked down in BT549 cells, CCK-8 test, and EdU assay were performed to measure the viability and growth of MDA-MB-468 and BT549 cells. Wound healing experiment was executed to determine the migration of BC cells. The invasion of cells was studied by the Transwell assay. Bioinformatics analysis, dual-luciferase reporter gene assay, qRT-PCR and Western blot were applied to predict and verify the binding sites between circ_0000518 and miR-1225-3p, miR-1225-3p and SOX4 mRNA. Pearson’s correlation analysis was utilized to evaluate the correlations among circ_0000518 expression, miR-1225-3p expression, and SOX4 mRNA expression in BC specimens. It was revealed that, circ_0000518 and SOX4 mRNA expression levels were up-modulated in BC tissues, while miR-1225-3p expression was down-modulated in BC tissues than that in adjacent tissues. Circ_0000518 overexpression or inhibition of miR-1225-3p remarkably enhanced the growth, migration as well as invasion of BC cells *in vitro*, whereas circ_0000518 knockdown or miR-1225-3p overexpression worked oppositely. Circ_0000518 was identified as a molecular sponge of miR-1225-3p, and it can up-regulate SOX4 mRNA expression via repressing miR-1225-3p. In conclusion, circ_0000518 is oncogenic in BC and functions through miR-1225-3p/SOX4 axis.

## Introduction

Breast cancer (BC) is one of the commonest malignancies among women and one of the main causes of cancer-related death worldwide [1]. Surgery, radiotherapy, and chemotherapy are the main treatment strategies for BC, but surgery is not optional for those patients with distant metastasis, and BC cells are often prone to show radioresistance and chemoresistance [2]. Searching for innovative and efficient treatments for BC is urgent, for which it is required to further clarify the molecular mechanism of BC progression [3].

Circular RNA (circRNA) has recently become a focus of cancer research. It is likely to become a new biomarker and therapeutic target for cancer, due to its expression specificity and structural stability [4,5]. Reportedly, some circRNAs participate in BC progression, and it often functions as competitive endogenous RNA (ceRNA) to regulate the biological processes. For instance, circ_0048764 is highly expressed in BC tissues, and promotes disease progression via modulating miR-1296-5p and TRIM14 [6]. Circ_0068033 enhances BC cell apoptosis by competitively binding with miR-659 [7]. Circ_0008039 facilitates BC cell growth, migration, and invasion by up-modulating CBX4 through sponging miR-515-5p [8]. Nonetheless, the role of circ_0000518 in BC are undefined.

MicroRNAs (miRNAs) are small non-coding (nc) RNA molecules [9]. MiRNAs are crucial modulators in cancer biology [10–13]. MiR-1225-3p is associated with several human diseases. For instance, a remarkable augmentation in miR-1225-3p expression can be detected in some cases of adrenal pheochromocytomas [14]. Nevertheless, neither the role nor the mechanism of miR-1225-3p in BC is defined.

Via analyzing the circRNA expression profile of BC tissues, we observed that circ_0000518 may be highly expressed in BC. We hypothesized that circ_0000518 was an oncogenic cricRNA in BC progression. The research is performed to elaborate on the role of circ_0000518 in BC. We report that, circ_0000518 has oncogenic properties in BC via serving as a ceRNA to modulate miR-1225-3p and Sry‑Related HMG box 4 (SOX4).

## Materials and methods

### Tissue specimens

Specimens of tumor tissues and paracancerous tissues (at least 3 cm from the margin of tumor tissues) from 43 BC patients obtained from the hospital were analyzed. None of the subjects had undergone preoperative anti-cancer treatment, such as radiotherapy or chemotherapy. In the surgery, all samples were instantly frozen in liquid nitrogen at −196°C. The research was authorized by the Ethics Committee of Dongying People’s Hospital, and all of the enrolled subjects signed an informed consent form before surgery.

### Cell culture and cell transfection

Human BC cell lines (BT549, MDA-MB-231, MDA-MB-468, MDA-MB-453) and normal mammary epithelial cells (MCF-10A) were obtained from American Type Culture Collection (ATCC, Manassas, VA, USA). These cells were cultured in RPMI1640 medium (Thermo Fisher Scientific, Waltham, MA, USA) containing 10% heat-inactivated fetal bovine serum (FBS;Thermo Fisher Scientific, Waltham, MA, USA) and routinely cultured at 37°C and 5% CO_2_. The cells were passaged every 2 to 3 d, and the cells in logarithmic phase were selected for the experiment.

After the MDA-MB-468 and BT549 cell lines were rinsed by sterile phosphate buffer saline (PBS), cell suspension was prepared. The cell density was moderated to 1 × 10^6^ cells/mL, and then the cells were inoculated into 12-well plates and cultured to 50–60% confluence. Then, the medium was replaced by serum-free medium, and after 12 h, cell transfection was executed using Lipofectamine® 2000 (Thermo Fisher Science, Waltham, MA, USA). 24 h later, the cells were cultured in completed medium. After another 24 h, the cells were harvested to validate the transfection efficiency. The circ_0000518 overexpression plasmid (circ_0000518), empty plasmid (NC), shRNA targeting circ_0000518 (sh-circ_0000518), and negative control shRNA (sh-NC), miR-1225-3p mimic and negative control for miR-1225-3p mimic, miR-1225-3p inhibitor and negative control for miR-1225-3p inhibitor were obtained from Riobio (Guangzhou, China).

### qRT-PCR

Total RNA was extracted with TRIzol reagent (Invitrogen, Carlsbad, CA, USA). Primer 6.0 was adopted to design the primers, which were synthesized by Takara (Dalian, China). The total RNA was reversely transcribed into cDNA as the template, and the PCR was executed with SYBR Premix Eaq^TM^ II (Takara, Dalian, China). MiR-1225-3p relative expression was normalized by U6, and circ_0000518 relative expression and SOX4 relative expression were normalized by GAPDH, and calculated using the 2^−ΔΔCt^ method. To determine the subcellular localization of circ_0000518 in BC cells, the nuclear RNA and cytoplasmic RNA of BC cells were, respectively, extracted by using a PARIS™ Kit (Thermo Fisher Scientific, Waltham, Mass., USA). Then, qRT-PCR was performed.

### CCK-8 method

2 × 10^3^ cells from each group were planted into each well of a 96-well plate. After the cells were cultured for 1, 2, or 3 d, 10 μL of CCK-8 (Beyotime Biotechnology, Shanghai, China) was supplemented to each well. The cells were then incubated for 4 h, and the absorbance value at 450 nm wavelength was measured by a microplate reader, with a well only containing the medium and CCK-8 solution without cells as the control.

### EdU experiment

BC cells were planted in 24-well plates (1.5 × 10^5^ cells/well). After the cells were cultured with serum-free medium for 24 h, a EdU Cell Proliferation Detection kit (Beyotime Biotechnology, Shanghai, China) was utilized to detect the cell growth following the instruction. After the cells were incubated with EdU solution for 2 h, the cells were fixed with 4% paraformaldehyde, stained using Click-It reaction solution. 0.5% Triton X-100 was used as an osmotic agent, and then DNA was counterstained with DAPI staining solution. After the cells were washed in PBS, the total number of fluorescence-labeled were counted under a fluorescence microscope.

### Wound healing experiment

The transfected cells were planted into in 6-well culture plates (2 × 10^6^ cells/well), and cultured until the cell confluence reach about 90%. Then, a perpendicular line was generated on the monolayer cells with a sterile pipette, and then the cells were rinsed 3 times with PBS to remove the floating cells. Subsequently, the cells were cultured with the medium containing 2.5% FBS. Then, the cell-free zone was observed, and the width was measured under a microscope at 0 h and 24 h, respectively. The closure of the cell-free zone was used to indicate the migration of BC cells.

### Transwell experiment

The transfected cells in serum-free medium were planted into the upper room of Transwell chambers (BD Biosciences, San Jose, CA, USA) that paved with a layer of Matrigel (BD Biosciences, San Jose, CA, USA) and the lower room of the chambers were added with medium containing 20% FBS. After 48 h of incubation, the medium was discarded, the cells in the top room were removed, and the cells which passed through the filter were fixed with methanol, and stained using 0.1% crystal violet for 15 min. After the cells were washed by PBS, under a microscope, for each chamber, five fields of view were randomly selected to count the cells, and the mean was calculated.

### Western blot

The RIPA lysis buffer (Beyotime Biotechnology, Shanghai, China) was supplemented to the cells and the total protein was extracted on the ice. After the samples were centrifuged, the supernatant was collected as the protein samples. BCA method was applied for protein quantification. Then, 5× loading buffer was mixed with the samples, and next the protein was denatured in boiling water. After SDS-PAGE, the protein was transferred on the PVDF membrane, and then the membrane was blocked in 3% bovine bull serum albumin (BSA) for 1 h. Then, the membranes were incubated with the primary antibodies including anti-SOX4 (ab243041, 1:1000, Abcam, Shanghai, China) and anti-GAPDH (ab8245, 1:3000, Abcam, Shanghai, China), respectively, overnight at 4°C. After the residual primary antibody on the membrane was rinsed off by TBST on a shaker, the membrane was incubated with HRP-labeled secondary antibody (goat anti-mouse IgG H&L, ab205719, 1:2500, Abcam, Shanghai, China) for 1 h at room temperature. After the residual secondary antibody on the membrane was washed off by TBST on the shaker, the Enhanced Chemiluminescence Western blotting Substrate (Dongguan Biotech, Shandong, China) was added on to the membrane to react with the protein-antibody complex, and the protein bands were developed. GAPDH was the internal reference.

### Dual-luciferase reporter gene assay

All luciferase reporter vectors, including circ_0000518 wild-type luciferase vector (circ_0000518 wt), circ_0000518 mutant luciferase vector (circ_0000518 mut), SOX4 3ʹUTR wild-type luciferase vector (SOX4 mRNA wt), and SOX4 3ʹUTR mutant luciferase vector (SOX4 mRNA mut) were constructed by Promega (Madison, WI, USA). MDA-MB-468 cells were planted in 48-well plates and cultured to 70% confluence. MDA-MB-468 cells were then co-transfected with circ_0000518 wt, circ_0000518 mut, SOX4 mRNA wt or SOX4 mRNA mut with miR-1225-3p mimics, miR-1225-3p inhibitors or control miRNAs. After 48 h of transfection, the luciferase activity of each well was determined, to examine the binding affinity between sequence.

### Statistical analysis

All tests were executed at least 3 times and the data were expressed as mean ± standard deviation (x ± s). The statistics analysis was performed with SPSS 23.0 (SPSS Inc., Chicago, IL, USA). One-way ANOVA with Turkey’s post-hoc test was employed for the comparison of differences among multiple groups, and an independent sample *t*-test was applied for the comparison between two groups, and a χ2 test was used for the comparison between two groups of counting data. *P* < 0.05 signified the statistical significance

## Results

In the present work, we investigate the expression characteristics of circ_0000518 in BC, and use gain-of-function cell model and loss-of-function cell model to investigate the biological function of circ_0000518 in BC cells. Bioinformatics analysis and molecular biological techniques were used to investigate the regulatory function of circ_0000518 on miR-1225-3p and SOX4.

### Circ_0000518 is abnormally up-moderated in BC tissues and cells

**Figure 1.**
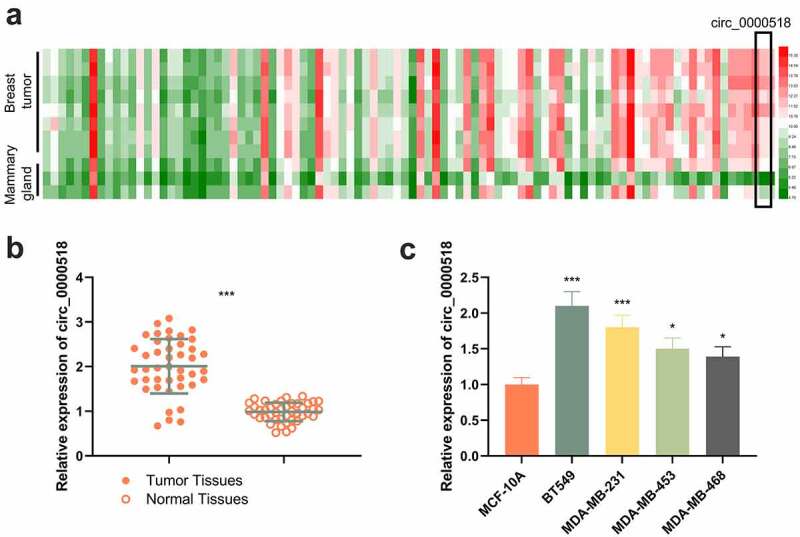
The expression characteristics of circ_0000518 in BC.

To search for the circRNAs that were abnormally expressed in BC, GSE101123 dataset was analyzed. This dataset contained circRNA expression profile of three cases of normal breast tissues and nine cases of BC tissues. It showed that circ_0000518 expression was notably up-moderated in BC tissues compare with normal breast tissues (Figure 1(a)). Additionally, in the collected BC specimens, circ_0000518 was markedly up-modulated in BC tissue compared with paracancerous tissue (Figure 1(b)). After dividing the BC patients into a circ_0000518 overexpression group (n = 21) and a circ_0000518 under-expression group (n = 22), it showed that circ_0000518 expression was markedly associated with tumor size, lymph node metastasis, TNM stage and tumor differentiation(Table 1). Moreover, circ_0000518 expression was augmented in BC cell lines relative to that in normal human breast epithelial cells (Figure 1(c)).

**Table 1.** Correlation between circ_0000518 expression level and characteristics of breast cancer patients (n = 43)

Feature	All patients (n)		Circ_0000518 expression level		Chi square value	*P* value
			Low expression	High expression		
All patients (n)	43		22	21		
Age (years)						
≤40		11	4	7	1.2956	0.255
>40		32	18	14		
Menopause						
Yes		23	10	13	1.1687	0.280
No		20	12	8		
Tumor size						
≤2 cm		18	13	5	5.4953	0.019*
>2 cm		25	9	16		
Lymph node metastasis						
No		19	14	5	6.9103	0.009**
Yes		24	8	16		
Clinical TNM stage						
I–II		25	16	9	3.9389	0.047*
III–IV		18	6	12		
Differentiation						
Well/moderate		29	18	11	4.2401	0.039*
Poor		14	4	10		

*p < 0.05, **p < 0.01.

(a) A heat map was used to present the significantly up-regulated circRNAs in BC tissues compared with normal breast tissues in the dataset GSE101123. (b) qRT-PCR was performed to detect circ_0000518 expression in BC tissues and paracancerous tissues. (c) qRT-PCR was used to detect circ_0000518 expression in normal mammary epithelial cells MCF-10A and BC cell lines BT549, MDA-MB-231, MDA-MB-453, MDA-MB-468.*, **, *** represent *P* < 0.05, *P* < 0.01, *P* < 0.001, respectively.

### Circ_0000518 enhances the growth, migration and invasion of BC cells

**Figure 2.**
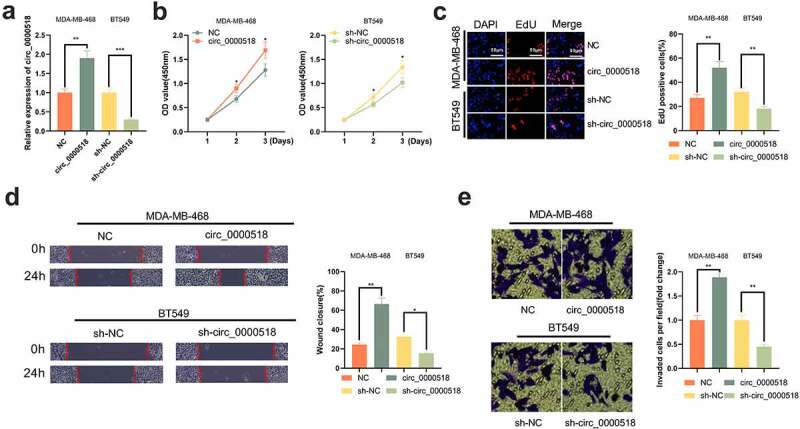
Biological role of circ_0000518 in BC cells.

Circ_0000518 overexpressing and knockdown cell lines were constructed using MDA-MB-468 cells and BT549 cells, respectively (Figure 2(a)). Subsequently, the effect of circ_0000518 on BC cells was explored. CCK-8 assay showed that the viability of MDA-MB-468 cell line with circ_0000518 overexpression were markedly enhanced; circ_0000518 overexpression led to more EdU-positive cells, also suggesting circ_0000518 promoted the proliferation of BC cells (Figure 2(b,c)). Additionally, wound healing assay and Transwell experiment revealed that circ_0000518 overexpression facilitated the migration and invasion of MDA-MB-468 cells (Figure 2(d,e)). The opposite phenomena was observed in the BT549 cell line with circ_0000518 knockdown. The data implied that circ_0000518 enhanced growth and aggressiveness of BC cells.

(a) qRT-PCR was performed to detect circ_0000518 expression in MDA-MB-468 and BT549 cells after the overexpression plasmids and siRNAs were transfected. (b) CCK-8 experiment was used to detect the changes in the viability of MDA-MB-468 and BT549 cells. (c) EdU assay was used to detect the changes in the growth of MDA-MB-468 and BT549 cells. (d) Scratch healing test was used to detect the migration of MDA-MB-468 and BT549 cells. (e) Transwell invasion assay was conducted to detect the changes in invasion capability of MDA-MB-468 and BT549 cells.*, ** represent *P* < 0.05, *P* < 0.01, respectively.

### Circ_0000518 specifically binds with miR-1225-3p

**Figure 3.**
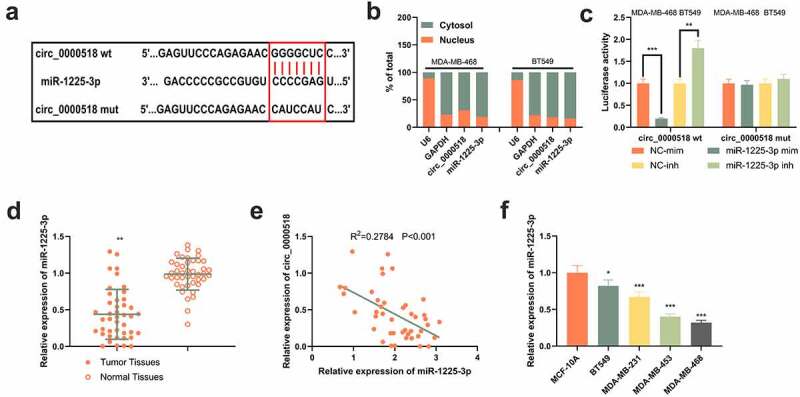
Circ_0000518 specifically targets and regulates miR-1225-3p.

To probe the mechanism by which circ_0000518 exerts its biological functions, the downstream targets of circ_0000518 were predicted using Circular RNA interactome database, and a potential binding sequence between miR-1225-3p and circ_0000518 was predicted (Figure 3(a)). To validate the binding relationship between the two, firstly, the distribution of circ_0000518 and miR-1225-3p in the cytoplasm and nucleus was examined, and both of them were predominantly expressed in the cytoplasm (Figure 3(b)). Furthermore, the prediction was validated using the dual-luciferase reporter gene test, which showed that miR-1225-3p overexpression markedly repressed the luciferase activity of circ_0000518 wt, while the inhibition of miR-1225-3p exerted the opposite effect, but neither transfection of miR-1225-3p mimics nor miR-1225-3p inhibitors induced significant changes in the luciferase activity of circ_0000518 mut (Figure 3(c)). These data suggested that circ_0000518 could target miR-1225-3p in BC cells. Additionally, miR-1225-3p expression in BC was detected. It was revealed that miR-1225-3p expression was markedly down-modulated in BC tissues compared with that in paracancerous tissues (Figure 3(d)). Furthermore, in BC samples, miR-1225-3p expression was negatively correlated with circ_0000518 expression (Figure 3(e)). Also, miR-1225-3p expression was lowly expressed in the BC cell lines as opposed to normal human breast epithelial cells MCF-10A (Figure 3(f)).

(a) The Circular RNA interactome database predicted that miR-1225-3p was the downstream target of circ_0000518. (b) Distribution of circ_0000518 and miR-1225-3p in the cytoplasm and nucleus of BC cells was examined by qRT-PCR. (c) Dual-luciferase reporter gene experiment was used to verify the binding relationship between circ_0000518 and miR-1225-3p. (d) qRT-PCR was conducted to detect miR-1225-3p expression in BC tissues and adjacent tissues. (e) Circ_0000518 was correlated with miR-1225-3p expression in BC tissues. (f) qRT-PCR was performed to detect miR-1225-3p expression in normal mammary epithelial cell line MCF-10A and BC cell lines BT549, MDA-MB-231, MDA-MB-453, MDA-MB-468.*, **, *** represent *P* < 0.05, *P* < 0.01, *P* < 0.001, respectively.

### Biological utility of miR-1225-3p in BC cell lines

**Figure 4.**
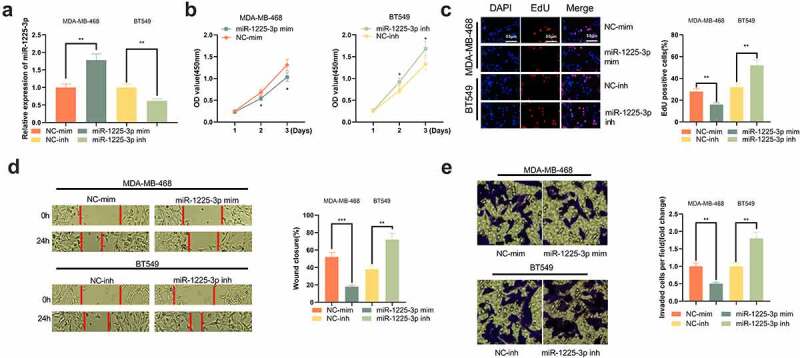
Biological function of miR-1225-3p in BC cells.

To elaborate on the effects of miR-1225-3p on BC cells, MDA-MB-468 cells were selected to construct a miR-1225-3p overexpression cell model, and BT549 cells were selected to construct a miR-1225-3p low-expression cell model (Figure 4(a)). It was revealed that, miR-1225-3p overexpression significantly suppressed the growth, migration and invasion of MDA-MB-468 cells, while transfection of miR-1225-3p inhibitors enhanced the growth, migration and invasion of BT549 cells (Figure 4(b–e)). The findings supported that miR-1225-3p had tumor-suppressive properties on BC cells.

(a) qRT-PCR was used to detect miR-1225-3p expression in MDA-MB-468 and BT549 cells after the transfection of miR-1225-3p mimics and inhibitors. (b) CCK-8 experiment was used to evaluate the changes in the viability of MDA-MB-468 and BT549 cells. (c) EdU assay was performed to detect the changes in the growth of MDA-MB-468 and BT549 cells. (d) Scratch healing test was used to detect the migration of MDA-MB-468 and BT549 cells. (e) Transwell invasion test was used to analyze the change of invasion ability of MDA-MB-468 and BT549 cells.*, ** represent *P* < 0.05, *P* < 0.01, respectively.

### miR-1225-3p counteracts the promoting effect of circ_0000518 on growth, migration and invasion of MDA-MB-468 cells

**Figure 5.**
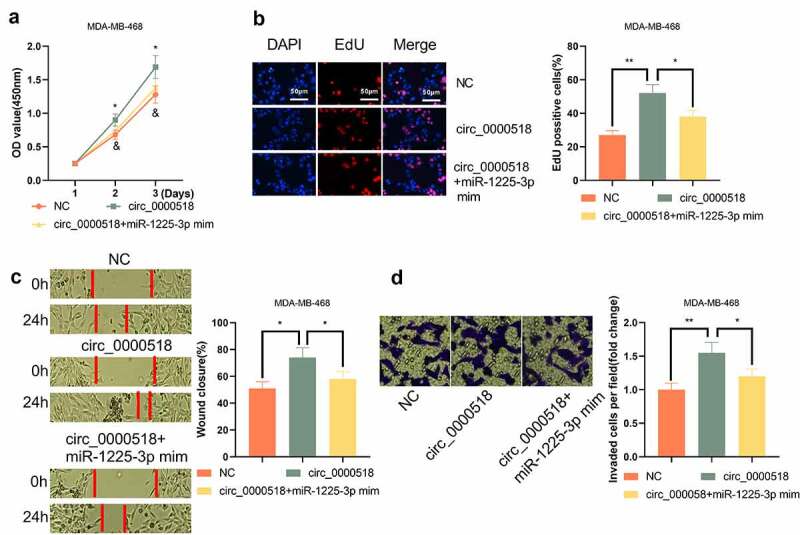
miR-1225-3p reverses the effect of circ_0000518 on MDA-MB-468 cells.

To verify whether circ_0000518 regulated the behaviors of BC cells through miR-1225-3p, circ_0000518 overexpression plasmid and miR-1225-3p mimics were co-transfected into MDA-MB-468 cells. It was revealed that, the regulatory effects of circ_0000518 overexpression on the viability, growth, migration and invasion of MDA-MB-468 cells were partially counteracted by the co-transfection of miR-1225-3p mimics (Figure 5(a–d)). The data of these experiments implied that, at least in part, circ_0000518 exerted its biological function in BC cells through repressing miR-1225-3p.

MDA-MB-468 cells were co-transfected circ_0000518 overexpression plasmids and miR-1225-3p mimics. (a) CCK-8 assay was employed to detect cell viability of MDA-MB-468 cells. (b) EdU experiment was used to measure the proliferative ability of MDA-MB-468 cells. (c) Scratch healing assay was used to detect the migration of MDA-MB-468 cells. (d) Transwell invasion assay was used to detect the changes in the invasion capability of MDA-MB-468 cells.*, ** represent *P* < 0.05, *P* < 0.01, respectively. In A * represents *p* < 0.05 for NC group compared to circ_0000518; & represents *p* < 0.05 for circ_0000518+ miR-1225-3p mim group relative to circ_0000518 group.

### Circ_0000518 represses SOX4 expression by targeting miR-1225-3p

**Figure 6.**
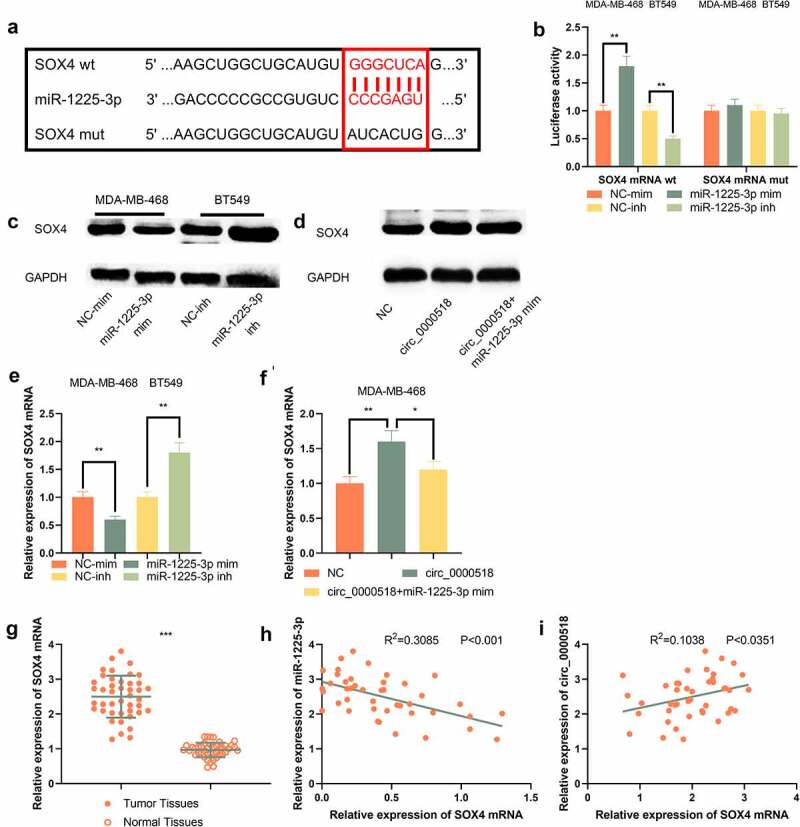
SOX4 expression is modulated by circ_0000518 and miR-1225-3p.

Next, The downstream mechanism of miR-1225-3p was further investigated. TargetScan database showed that miR-1225-3p had the binding site to the 3ʹUTR of SOX4 mRNA (Figure 6(a)). Additionally, the dual-luciferase reporter gene experiment was applied for validation of the binding site, which indicated that the luciferase activity of SOX4 mRNA wt was suppressed after transfection with miR-1225-3p mimics, while the luciferase activity of SOX4 mRNA wt was increased after transfection with miR-1225-3p inhibitors, but the selective regulation of miR-1225-3p had no remarkable function on the mutated reporters (Figure 6(b)). SOX4 mRNA expression was revealed to be notably higher in BC tissues than in adjacent tissues (Figure 6(c)), and miR-1225-3p was negatively correlated with SOX4 mRNA expression (Figure 6(d)) and circ_0000518 was positively correlated with SOX4 mRNA expression (Figure 6(e)). Importantly, the transfection of miR-1225-3p mimics could down-modulate the expression levels of SOX4 protein and mRNA; besides, circ_0000518 exerted the opposite effect, while miR-1225-3p restoration partially counteracted the up-modulation of SOX4 induced by circ_0000518 overexpression (Figure 6(f–i)). The above data indicated that circ_0000518 up-modulated SOX4 expression at protein and mRNA levels by down-modulating miR-1225-3p.

(a) The TargetScan database predicted the presence of binding sites between SOX4 mRNA 3ʹUTR and miR-1225-3p. (b) Dual-luciferase reporter gene experiment was validated for the binding site between SOX4 mRNA and miR-1225-3p. (c) Western blot was used to detect the changes of SOX4 expression in MDA-MB-468 and BT549 cells after miR-1225-3p was up-regulated or inhibited. (d) Western blot experiment was used to detect the changes of SOX4 expression in MDA-MB-468 cells after MDA-MB-468 cells were transfected with circ_0000518 overexpression plasmids or co-transfected with circ_0000518 overexpression plasmids and miR-1225-3p mimics. (e, f) After transfection, qRT-PCR was used to detect SOX4 mRNA expression in MDA-MB-468 and BT549 cells. (g) qRT-PCR was used to detect SOX4 mRNA expression in BC tissues and paracancerous tissues. (h) Correlation between miR-1225-3p expression and SOX4 mRNA expression in BC tissues. (i) Correlation between circ_0000518 expression and SOX4 mRNA expression in BC tissues.*, **, *** represent *P* < 0.05, *P* < 0.01, *P* < 0.001, respectively.

## Discussion

Although the role of circRNA in tumorigenesis is gradually unveiled, the biological function of a lot of circRNAs has not been reported [15]. Recent studies report that certain circRNAs are dysregulated in BC tissues, and participate in disease progression [16]. For instance, circNFIC represses miR-658 and thereby modulates UPK1A expression to impede BC cell growth and migration [17]. CircIQCH is up-modulated in BC tissues and enhances the growth and migration of BC cells through the miR-145/DNMT3A axis [18]. The role of circ_0000518 in tumors is undefined, and in the present work, the up-moderation of circ_0000518 expression was observed in cancer tissues of BC patients, which was associated with unfavorable pathological indicators. Functionally, circ_0000518 facilitates the growth, migration and invasion of BC cells. The data imply that circ_0000518 may be a novel potential biomarker and therapy target for BC.

MiRNAs are crucial modulators in diverse cancers’ progression [11]. Reportedly, miR-1225-3p overexpression is associated with adverse prognosis in biliary tract cancer [19]. The present work probed the function of miR-1225-3p in BC cells and revealed that miR-1225-3p was down-regulated in BC, and it repressed the growth, migration and invasion of BC cells. ceRNA mechanism is a crucial path by which non-coding RNAs exerts their biological functions [20]. For instance, circ_0000526 restrains BC cell growth, migration and invasion by targeting and repressing miR-492 expression and enhancing SOCS2 expression [21]. Circ_0007255 modulates BC progression through the miR-335-5p/SIX2 axis [22]. CircMMP11 enhances the malignant biological behaviors of MDA-MB-231 cells by decoying miR-1204 [23]. In this work, the binding sequence between circ_0000518 and miR-1225-3p was verified; in addition, it was revealed the promoting function of circ_0000518 on growth, migration and invasion of MDA-MB-468 cells was partially counteracted by miR-1225-3p. This work reveals that miR-1225-3p is specifically modulated by circ_0000518, partly explaining the mechanism of the dysregulation of miR-1225-3p in BC.

SOX4 is a transcription factor of the SOX (SRY-associated HMG-box) family with a highly conserved HMG-box DNA-binding domain [24]. Reportedly, SOX4 is vital in the modulation of growth, migration, and invasion of tumor cells [25]. For instance, SOX4 is validated to accelerate the growth, migration and invasion of colorectal cancer cells [26]. SOX4 facilitates the growth and impedes the apoptosis in prostate cancer cells [27]. In BC, SOX4 enhances the growth, migration, invasion, and suppresses the apoptosis of BC cell lines MDA-MB-231 and MCF-7 [28]. SOX4 overexpression is also reported to be an unfavorable prognostic biomarker for BC patients [29]. Mechanistically, SOX4 can work as a driver of PI3K/Akt signaling to facilitate BC development [30]. In this work, circ_0000518 was unveiled to indirectly up-modulate SOX4 expression by specifically down-modulating miR-1225-3p, thereby accelerating the growth, migration and invasion of BC cells. The data suggest that circ_0000518 may regulate PI3K/Akt signaling to participate in BC development via modulating miR-1225-3p/SOX4 axis, which requires further investigation in the following work.

## Conclusion

In summary, the work reports that circ_0000518 is up-modulated in BC tissues and cells and can indirectly up-modulate SOX4 expression by specifically repressing miR-1225-3p to facilitate the growth, migration and invasion of BC cells. The research brings novel ideas for further screening of molecular targets that may contribute to BC therapy.

## Research highlights

1. A targeting relationship between circ_0000518 and miR-1225-3p in breast cancer is identified.

2. A targeting relationship between miR-1225-3p and SOX4 in breast cancer is identified.

3. Circ_0000518, miR-1225-3p and SOX4 form a ceRNA network to promote breast cancer progression.
